# Occurrence and diversity of stem nodulation in *Aeschynomene* and *Sesbania* legumes from wetlands of Madagascar

**DOI:** 10.1038/s41598-024-55247-7

**Published:** 2024-02-29

**Authors:** Faustin F. Manantsoa, Marrino F. Rakotoarisoa, Clémence Chaintreuil, Adamson T. E. Razakatiana, Frédéric Gressent, Marjorie Pervent, Mickaël Bourge, Martial D. Andrianandrasana, Nico Nouwen, Herizo Randriambanona, Heriniaina Ramanankierana, Jean-François Arrighi

**Affiliations:** 1 Laboratoire de Microbiologie de l’Environnement-Centre National de Recherches sur l’Environnement, BP 1739, Fiadanana, Antananarivo Madagascar; 2 Department of Ethnobotany and Botany, National Center for Applied Pharmaceutical Research, Antananarivo 101, Madagascar; 3 Plant Health Institute of Montpellier (PHIM), University Montpellier/IRD/INRAE/CIRAD/SupAgro, Campus de Baillarguet, 34398 Montpellier, France; 4 Cytometry Facility, Institute for Integrative Biology of the Cell (I2BC), Imagerie-Gif, Université Paris-Saclay, CEA, CNRS, 91198 Gif-Sur-Yvette, France

**Keywords:** Plant sciences, Plant symbiosis, Rhizobial symbiosis

## Abstract

Legumes have the ability to establish a nitrogen-fixing symbiosis with soil rhizobia that they house in specific organs, the nodules. In most rhizobium-legume interactions, nodulation occurs on the root. However, certain tropical legumes growing in wetlands possess a unique trait: the capacity to form rhizobia-harbouring nodules on the stem. Despite the originality of the stem nodulation process, its occurrence and diversity in waterlogging-tolerant legumes remains underexplored, impeding a comprehensive analysis of its genetics and biology. Here, we aimed at filling this gap by surveying stem nodulation in legume species-rich wetlands of Madagascar. Stem nodulation was readily observed in eight hydrophytic species of the legume genera, *Aeschynomene* and *Sesbania*, for which significant variations in stem nodule density and morphology was documented. Among these species, *A. evenia*, which is used as genetic model to study the rhizobial symbiosis, was found to be frequently stem-nodulated. Two other *Aeschynomene* species, *A. cristata* and *A. uniflora*, were evidenced to display a profuse stem-nodulation as occurs in *S. rostrata*. These findings extend our knowledge on legumes species that are endowed with stem nodulation and further indicate that *A. evenia*, *A. cristata*, *A. uniflora* and *S. rostrata* are of special interest for the study of stem nodulation. As such, these legume species represent opportunities to investigate different modalities of the nitrogen-fixing symbiosis and this knowledge could provide cues for the engineering of nitrogen-fixation in non-legume crops.

## Introduction

The symbiosis between legume plants and soil rhizobia results in the formation of nitrogen-fixing nodules, generally exclusively appearing on the roots. However, in a handful of tropical legumes growing in wetlands, nodulation with rhizobia can also occur at stem-located dormant root primordia, a process that is referred to as stem nodulation. Given the semi-aquatic lifestyle of these legumes, it has been hypothesized that the stem nodulation trait is an evolutionary adaptation to flooding^1,2^. Stem nodulation was first described for the African *Aeschynomene afraspera* (not to be confounded with *Aeschynomene aspera* found in Asia)^3^. Stem nodulation gained agricultural interest after the discovery that the profuse stem nodulation as found in *A. afraspera* and *Sesbania rostrata* results in a high nitrogen fixation activity^4–6^. To date, stem nodulation has been reported for species belonging to four legume genera: *Aeschynomene*, *Discolobium*, *Neptunia* and *Sesbania*^7^. While, in the three latter genera, stem-nodulation has been described for one or very few species, more than 20 *Aeschynomene* species have been shown to form stem nodules^8,9^. These legume species can differ in their stem nodulation ability and intensity, and to date *S. rostrata* and *A. afraspera* are the only ones shown to have a very profuse stem-nodulation^7^.

Strikingly, *S. rostrata* is stem-nodulated only by *Azorhizobium caulinodans*^10,11^. While the water-tolerant but root-nodulating *Sesbania virgata* also interacts with an *Azorhizobium* species, *A. doebereinerae*, other *Sesbania* species root-nodulate with rhizobia of either the *Ensifer* (formerly *Sinorhizobium*), *Mesorhizobium* or *Rhizobium* genera^12,13^. Similarly, different types of *Bradyrhizobium* strains have been identified as nodulating *Aeschynomene* species^7,14^. The most important difference between the strains were the presence or absence of photosynthetic activity and *nod* genes to produce Nod factors^15–17^. So far, photosynthetic *Bradyrhizobium* strains have been exclusively found in nodules of stem-nodulating *Aeschynomene* species^7^ and strains lacking *nod* genes have been isolated from nodules of *Aeschynomene* species that cluster in a single clade, hereafter referred as the Nod-independent clade^8,18,19^. The *Bradyhrizobium* ORS278-*A. evenia* interaction serves as model for the deciphering of this very specific interaction called Nod-independent symbiosis^20,21^. In contrast, strains having *nod* genes, such as *Bradyrhizobium* ORS285*,* have been isolated from nodules of *A. afraspera* that is one of the *Aeschynomene* species using a Nod-dependent interaction^16^. The *S. rostrata* stem-nodulating *A. caulinodans* is not photosynthetic and has *nod* genes, but in both cases, the symbiotic interaction is very specific.

Although research has shed some light on the genetics of both partners in the *Bradyrhizobium*-*Aeschynomene* and *A. caulinodans*-*S. rostrata* symbiotic systems, our understanding of the molecular mechanism that causes stem nodulation is still in its infancy. Furthermore, our knowledge of the diversity and occurrence of stem nodulation *in natura* is relatively limited as it has been investigated in only a few geographical regions^15,17,22^. To fill this gap, we conducted a field study in Madagascar that contains a variety of wetlands with an important plant biodiversity. Here, we report on the stem-nodulated *Aeschynomene* and *Sesbania* legume species from Malagasy wetlands, by providing detailed information on their stem nodule density and morphology as observed in the field. Among the eight stem-nodulated species identified are *A. evenia*, which is used as model for the study of the Nod-independent symbiosis, and *A. cristata* and *A. uniflora* whose profuse stem nodulation represent new observations. We discuss the opportunity of these resources to fuel research on the nitrogen-fixing symbiosis in legumes and for the engineering of nodulation in non-legume crops.

## Results

**Figure 1:**
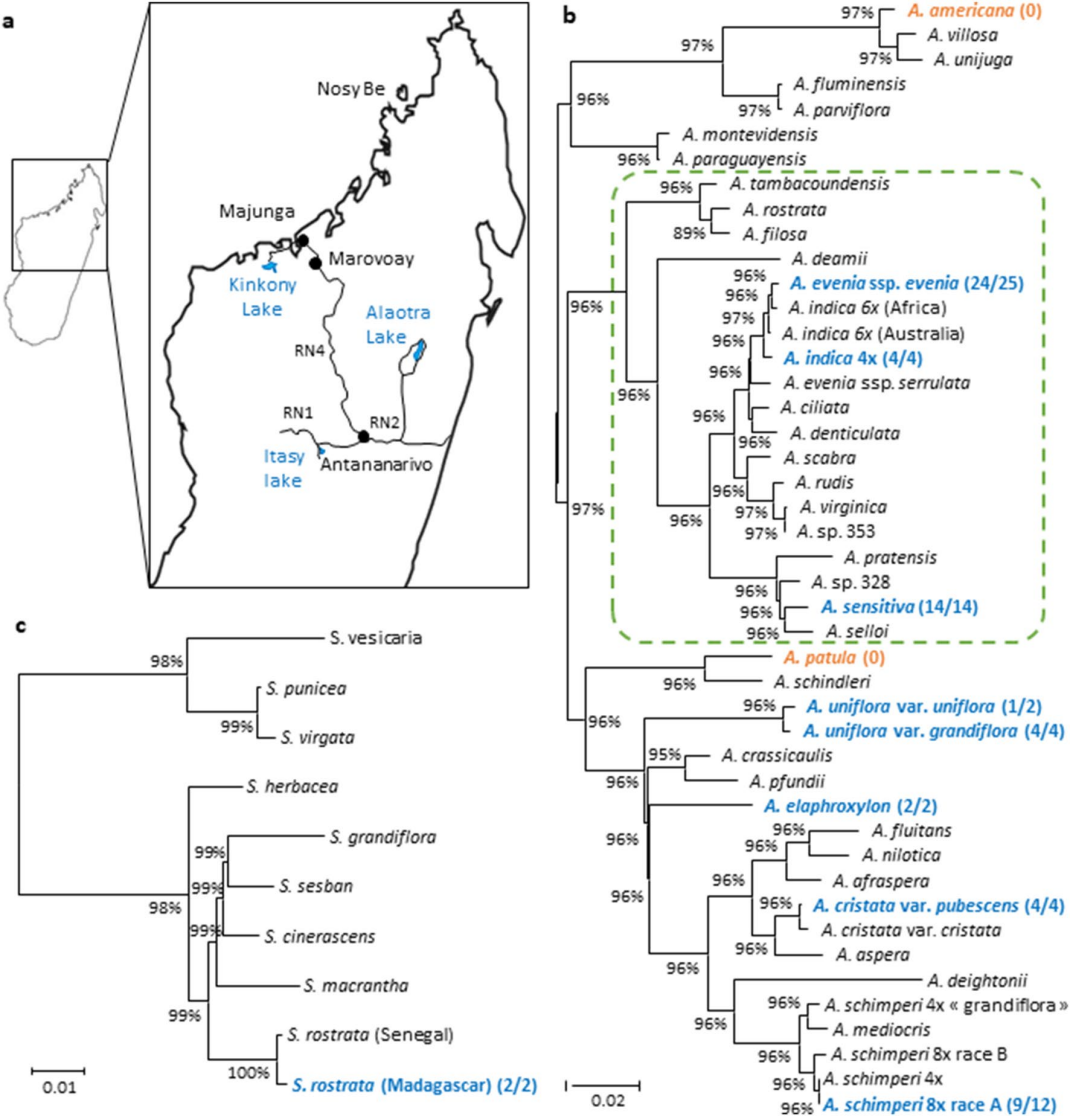
*Aeschynomene* and *Sesbania* species collected in wetlands of Madagascar. (**a**) Map of Madagascar with a zoom on the four collecting sites, including RN1-Itsay Lake, RN2-Alaotra Lake, RN4-Majunga and Nosy Be. (**b**) Phylogeny of *Aeschynomene* species. (**c**) Phylogeny of *Sesbania* species. In (**b**) and (**c**), Maximum likelihood phylogenetic reconstructions were obtained using the concatenated *ITS* + *matK* sequences. Numbers at nodes represent bootstrap values (% of 1000 replicates). Dashed boxes delineate clades where stem nodulation has been reported to occur. Taxa collected in Madagascar are in bold and numbers on their right correspond to the occurrence of stem nodulation in the different collection sites. In orange: no stem nodulation observed, in blue: stem nodulation observed in the present study.

A series of expeditions were organized to explore wetland-rich regions in the Central (RN1-Itasy Lake), Northern (Nosy Be), Western (RN4-Majunga) and Eastern (RN2-Alaotra Lake) parts of Madagascar (Fig. 1a). In these regions, we found stem-nodulated *Aeschynomene*, *Sesbania* and *Neptunia spp*. These latter were omitted from this analysis, as nodules were only found on floating stems forming adventitious roots and they tend to be considered as adventitious root nodules rather than genuine stem nodules. So, focusing on *Aeschynomene* and *Sesbania spp*, a total of 69 samples were collected (Supplementary Table 1). Based on the plant morphologies, these samples were assigned to eight species (Supplementary Table 1). Field observations were complemented with flow cytometry and molecular analyses for several collected plant specimens (Supplementary Tables 2 and 3). Genome size determination along with the sequencing of both the nuclear *ITS* region (700 nucleotides) and the chloroplast *matK* gene (1834 nucleotides) allowed an accurate specimen identification and investigation of the infra-specific diversity (Supplementary Figs. S1–S4). For each of the *Aeschynomene* and *Sesbania* genera, the information on the *ITS* and *matK* sequences was combined. Using this method, we were able to position all different Malagasy taxa for which the stem-nodulation is observed in the field in a single phylogenetic tree (Fig. 1b,c).

### Stem nodulation in Nod-independent *Aeschynomene* species

**Figure 2:**
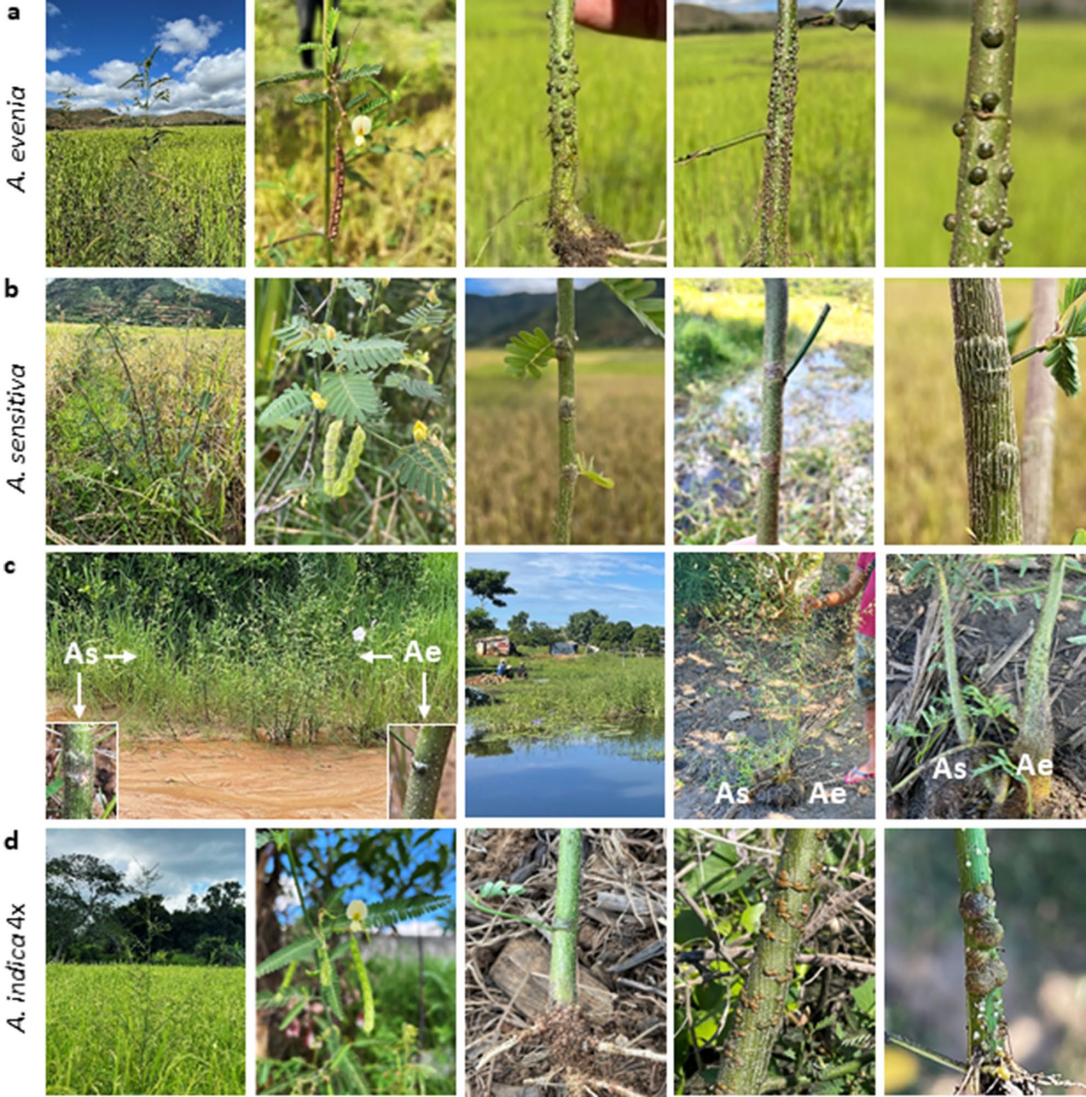
Stem nodulation in *Aeschynomene* species of the Nod-independent clade. (**a**) *A. evenia*. From left to right: 1- plant growing in a ricefield near Camp Bandro (Alaotra lake), 2- axillary axis bearing a yellow flower and a mature pod, 3–4- stem nodules located at the base or middle portion of the stem, 5- morphology of stem nodules. (**b**) *A. sensitiva*. From left to right: 1- plant growing in a rice field at Manakambahiny, in the direction of Alaotra lake, 2- axillary axis bearing yellow flowers and developing pods, 3–4- more or less flattened stem nodules, 5- morphology of collar stem nodules. (**c**) Co-occurrence of *A. evenia* and *A. sensitiva*. From left to right: 1- Stand of a mixed population in a shallow sandy river at Marofotroboka (next to RN4), insets show stem nodules of *A. sensitiva* and *A. evenia*, respectively, 2–4- permanent marsh at Belobaka (Majunga) where *A. sensitiva* and *A. evenia* where found to grow side-by-side with entangled roots, both plants displaying stem nodules. Ae: *A. evenia*, As: *A. sensitiva*. (**d**) *A. indica* 4x. From left to right: 1- plant growing in a rice field in Nosy Be, 2- axillary axis bearing a yellow flower and developing pods, 3–4- stem nodules located near the base or at the middle portion of the stem, 5- morphology of stem nodules. Note the presence of numerous pink root nodules in 3.

Three stem-nodulated *Aeschynomene* species belonging to the Nod-independent clade were found in Malagasy wetlands (Fig. 1b). *A. evenia* was by far the most widespread one, being seen in all parts of Madagascar and in all wetland types: river banks, marshes and rice fields. *A. evenia* has a transatlantic distribution and a well-defined geographically-structured genetic diversity^18^. The form present in Madagascar was previously classified as the Eastern African genotype. As a result, it is closely related to the reference line from Malawi that was selected for the genetic dissection of the Nod-independent symbiosis^21^. Stem-nodulated *A. evenia* plants were observed in 24 out of the 25 sampling sites. Nodules were green, indicative of the presence of chloroplasts, and often located in the lower part of the stem, but their distribution could extend to the upper branch-containing part and they were usually present in a profuse fashion. These stem nodules were hemispherical and with a broad attachment to the stem (Fig. 2a).

The second typical species found in different wetlands is *A. sensitiva*. Similarly to *A. evenia*, this species has also a transatlantic distribution and the genotype occurring in Madagascar is concommitantly present in Africa and Eastern Brazil^18^. *A. sensitiva* is well-known because the model strain ORS278, a photosynthetic *nod* gene-lacking *Bradyrhizobium*, was isolated from stem nodules in Senegal^16,23^. The photosynthetic activity of this strain was demonstrated to be important for the efficiency of stem nodulation. In addition, *A. sensitiva* has the particularity to develop unique ‘collar’ nodules on the stem (Fig. 2b). These were green and readily observed in all 14 sampling sites. Although *A. sensitiva* was globally less frequently found as compared to *A.evenia*, at the collection sites the two species were frequently growing adjacent to each other (Fig. 2c). The two species are known to have overlapping distributions in Madagascar but *A. sensitiva* is absent in the drier regions^24^.

Unexpected was the discovery of *A. indica* in marshes from Majunga and in rice fields in Nosy Be because this species is not native of Madagascar. *A. evenia* and *A. indica* are morphologically similar but they can be distinguished from each other via the flowers that on *A. indica* plants are in general larger than those on *A. evenia* (Fig. 2d). This high resemblance is due to their belonging to a same polyploid species complex where *A. evenia* 2x, *A. indica* 4 × and *A. indica* 6 × forms are present^18,25^. To confirm the visual species identification, we sequenced the nuclear *ITS* and chloroplastic *matK* gene for the specimens JFA29 and JFA109. Both their *ITS* and *matK* sequences were found to be 100% identical to other previously genotyped *A. indica* 4 × plants (Supplementary Fig. 1)^25^. We also determined the genome size of these two specimens, whose values were typical of *A. indica* 4 × plants (Supplementary Table 2)^25^, thus providing further support to the visual identification. In the four collection sites, *A. indica* plants were stem-nodulated, the nodules varying in shape and size but always with an enlarged base and with a green color (Fig. 2d). Similar to other *Aeschynomene* species, pink root nodules could be observed in unflooded conditions, (Fig. 2d).

### Stem nodulation in Nod-dependent *Aeschynomene* species

Among *Aeschynomene* species falling outside of the Nod-independent clade, the pantropical *A. americana* and the endemic *A. patula* were frequently present in explored sites. Both species are of interest because they have been proposed as model plants complementary to *A. evenia* to develop a comparative genetic system to study the Nod-independent and Nod-dependent symbioses in *Aeschynomene*^19^. However, for both species no stem-nodulated plants were found at the collection sites (Fig. 1b).

**Figure 3:**
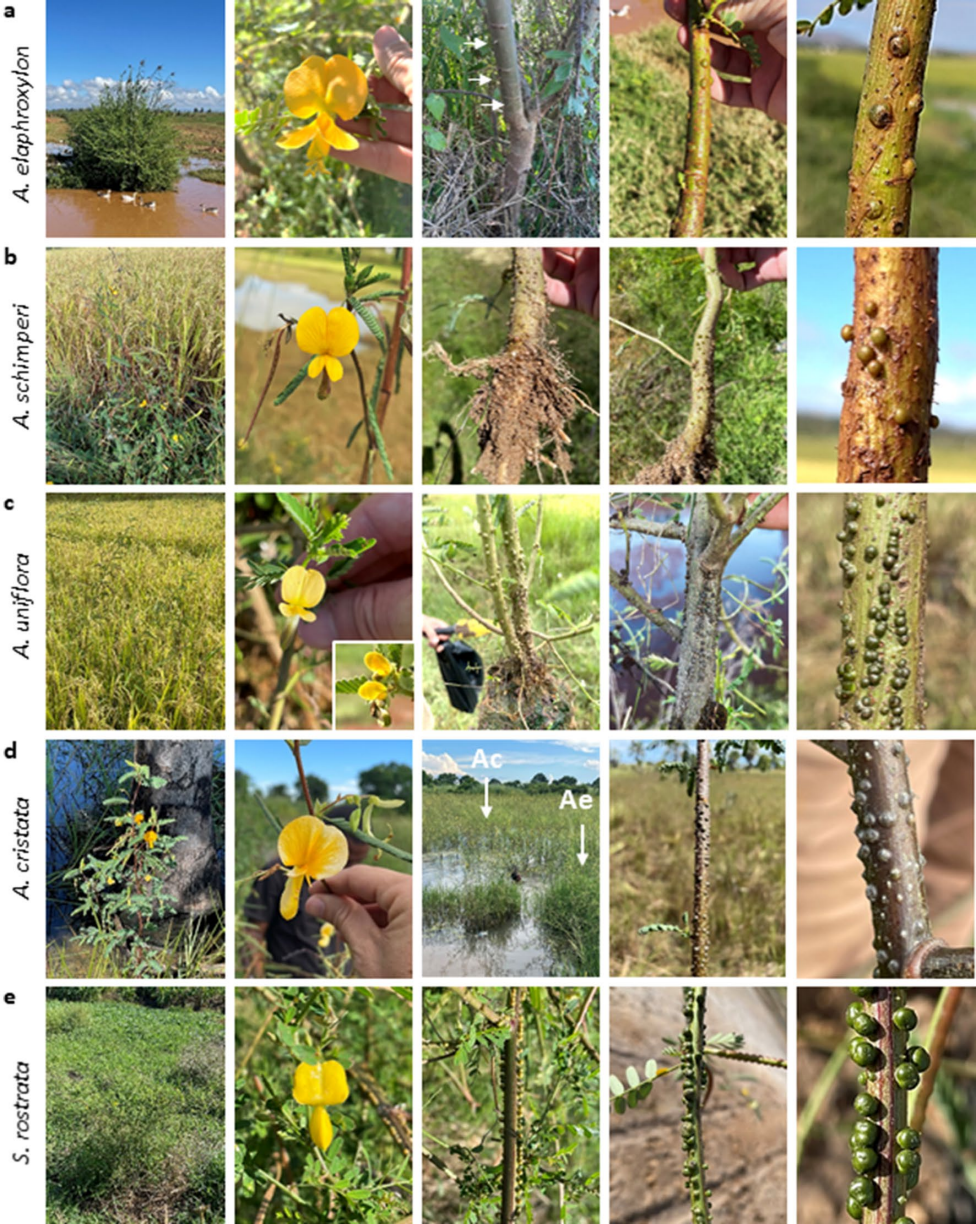
Stem nodulation in Nod-dependent *Aeschynomene* and *Sesbania* species. (**a**) *A. elaphroxylon*. From left to right: 1- stand of shrubby plants growing at the edge of a marsh near the Alaotra lake, 2- « showy » yellow flower with visible anthers, 3- woody stem with spines, 4- nodules developing at the base of the stem, 5- morphology of stem nodules. (**b**) *A. schimperi*. From left to right: 1- young plants in a ricefield at Maharefo, South of the Alaotra lake, 2- yellow flower, 3- pink root nodules, 4- stem nodules, 5- morphology of stem nodules. (**c**) *A. uniflora*. From left to right: 1- plants growing in a ricefield at Bongomena next to the RN4, 2- yellow flowers with the smaller variant form shown in the inset, 3 and 4- stem nodules of the of the normal and morphological variant, respectively, 5- morphology of stem nodules. (**d**) *A. cristata*. From left to right: 1- Plant growing in a swampy palm grove, 2- showy yellow flower, 3- Stand of *A. cristata* (Ac) in the center of a marsh lined with *A. evenia* (Ae), 4- nodules present all along the stem, 5- morphology of stem nodules. (**e**) *S. rostrata*. From left to right: 1- plant growing on the edge of a marsh at Amboromalandy next to the RN4, 2- Yellow flower, 3 and 4- arrows of nodules running all along on the stem, 5- morphology of stem nodules.

Two *Aeschynomene* species, *A. elaphroxylon* and *A. schimperi,* which are believed to be introduced in Madagascar, were found in the Central Plateaux (Fig. 1b)^24^. *A. elaphroxylon* was specially present around the Aloatra lake. It is a distinctive *Aeschynomene* species as it can form large shrubs to small trees with very showy yellow flowers and spiny stems (Fig. 3a). In the two sampled sites, plants were scarcely nodulated and, if so, only on the submerged parts of the stems. In that case, nodules were green and had a flattened hemispherical shape with a broad attachment to the stem (Fig. 3a). In contrast, *A. schimperi* was frequently found in rice fields. For this species, previous genetic analysis uncovered the presence of 4 × and 8 × cytotypes, and Malagasy specimens correspond to the 8 × cytotype^9^. In non-waterlogged conditions, numerous pink nodules could be observed on the main root, while under flooded conditions (9 of the 12 sampled sites) green nodules were present in the lower part of the stem (Fig. 3b). These nodules were spherical with a narrow attachment to the stem.

In regions at lower altitude, in the North and West of Madagascar, two other Nod-dependent *Aeschynomene* species were found. *A. uniflora* was present in the region of Majunga and in Nosy Be. Here it grew either at the edge of marshes or in rice fields where often also *A. evenia* or *A. sensitiva* species were present. Strikingly, two plant morphotypes were observed: one corresponding to erect plants with prominent flowers (3 sampled sites), and a second represented by shrubby plants bearing small flowers (two sampled sites) (Fig. 3c). It is likely that the two morphotypes correspond to the botanical varieties *A. uniflora* var. *grandiflora* and var. uniflora (Fig. 1b)^26^. *ITS* sequencing for the samples JFA51 and JFA71 (examples of plants with prominent and small flowers, respectively) revealed differences in their sequences (5 single nucleotide polymorphisms (SNPs)) that seem to discriminate large flower *A. uniflora* accessions (including JFA51) from small flower-bearing ones presently and previously characterized (JFA71, LSTM61, LSTM137) (Supplementary Fig. 2)^9^. Variations in the *matK* sequences were also observed but these were not correlated with the flower type (Supplementary Fig. 2). Moreover, flow cytometry measurements were relatively homogeneous (, Supplementary Table 2)^9^. In both morphotypes, numerous green nodules with a spherical shape and a narrow neck, running on the stem were visible (Fig. 3c).

*A. cristata* was found in pristine marshes and slow-flowing streams between Majunga and Mitsinjo where they often formed important stands of flooded plants. At sites where *A. cristata* plants were found *A. evenia* plants could be also present (Fig. 3d). *A. cristata* has attracted little attention until it was shown to be one of the genome donors of *A. afraspera* and being a sister species of the Asian *A. aspera*, both species being profusely stem nodulated^8,9,27,28^. Whereas the previously characterized *A. cristata* specimen (LSTM169) had densely-hairy stems^9^, those found and collected in Madagascar (including JFA34) were glabrous. Based on this characteristic, they may be tentatively associated to *A. cristata* var. *cristata* (hairy stems) and var. *pubescens* (glabrous stems) as previously described (Fig. 1b)^26^. However, *ITS* and *matK* sequencing, along with flow cytometry, revealed very little variations between both accessions (2 SNPs in the *ITS* and 3 SNPs in the *matK* sequences) (Supplementary Fig. 3, Supplementary Table 2). Thus, to assess any intra-specific genetic differentiation within the collected *A. cristata* accessions additional data are required. In all sampling sites, *A. cristata* specimens caught attention very quickly due to the profuse nodulation all over the stem (Fig. 3d). These stem nodules were green and hemispherical with a broad base as described for the related *A. afraspera* species^29^.

### Stem nodulation in *Sesbania rostrata*

*S. rostrata* was observed in only two sampling sites in the region of Majunga. *S. rostrata* has been used a research model for nodulation due to its profuse stem nodulation and its ability to switch from classical nodulation to Lateral Root Base nodulation in flooded conditions^5,30^. *S. rostrata* is the single species of its lineage in the *Sesbania* phylogeny^31^. However, *S. rostrata* specimens of Senegal and Madagascar were shown to be morphologically different and both hybridization and grafting experiments were less successful when interspecific^32^. For this reason, it has been proposed they could represent different subspecies of *S. rostrata* (Fig. 1c)^32^. *ITS* and *matK* sequencing of *S. rostrata* samples JFA21 and JFA45 point to a possible genetic differentiation when compared to accessions from Senegal with 5 SNPs in the *ITS* and a single SNP in the *matK* sequences, while flow cytometry analysis revealed similar genome sizes for the same accessions (Supplementary Fig. 4, Supplementary Table 2). In plants of both sampling sites, nodulation all along the stem was remarkable for its profusion and nodulation sites were typically distributed in vertical rows (Fig. 3e). These stem nodules were green, prominent, and had a constricted base.

## Discussion

In 2001, James et al. identified several stem-nodulated legumes in the Brazilian Pantanal wetlands^22^. To broaden our knowledge on stem nodulating legumes, this research inspired us to explore wetlands in a geographically distinct tropical region, Madagascar. In Malagasy wetlands, a large occurrence and diversity of stem nodulation in *Aeschynomene* and *Sesbania* legumes was detected. While some of the species found are already well-known to form stem nodules (*A. elaphroxylon*, *A. evenia*, *A. indica* 4x, *A. schimperi*, *A. sensitiva* and *S. rostrata*), for two other identified species, *A. cristata* and *A. uniflora*, this has been once mentioned in a review article, as unpublished observations, and subject to very limited experimentations in greenhouse conditions^1,9^. Here, we report on their stem nodulation in the field. Following the traditional classification of stem-nodulating legumes, *A. cristata* was convincingly a *bona fide* profusely stem-nodulated species, equalling the stem nodulation level found for *A. afraspera* and *S. rostrata*^1,7^. *A. uniflora* stem nodulation did not reach such level in the field. However, in greenhouse conditions *A. uniflora* stem nodulation has been shown to be exceptionally dense and to occur all along the stem^9^, indicating a profuse stem-nodulation capacity. The discovery of all these species and the demonstration of genetic diversity in several of them (*A. cristata*, *A. uniflora*, and *S. rostrata*) point out that more systematic studies including the collection of plants to evaluate species and ecotypes endowed with stem-nodulation are required.

What lessons could we learn from these studies? On the plant side, profuse stem nodulation may be indicative of high nitrogen fixation activity as previously demonstrated for *A. afraspera* and *S. rostrata*^4,5^. In these species, a nitrogen-fixing activity of stem nodules is less inhibited by assimilable N-sources in the soil^4,5^. Secondly, the presence of two stem-nodule morphologies (either hemispherical with a broad attachment to the stem or spherical with a narrow base) supports the existence of two developmental programs. In both cases the photosynthetic activity in stem nodules (inferred from their green colour for the specimens analysed in the present work) points to a physiology that likely differs from those of root nodules^33–35^. This photosynthetic activity in stem nodules is also of importance as it can contribute to energy supply to sustain the rhizobial nitrogen-fixing activity^1,7^. On the bacterial side, while *A. caulinodans* has been isolated from *S. rostrata* stem nodules in both Senegal and Madagascar^10,11^, a great variety of *Aeschynomene*-nodulating *Bradyrhizobium* strains do exist but their genetics are insufficiently understood^15,17,36^. Some of them are quite original in having a photosynthetic activity and using light wavelengths that are not used by the plant, meaning that stem nodule bacteroids can also produce energy for nitrogen fixation^23,33^. New photosynthetic strains having or lacking *nod* genes are expected to be present in stem nodules of *A. cristata* (because it is a parent species of *A. afraspera*) and of the Nod-independent *Aeschynomene* species (e.g. *A. evenia*, *A. indica* 4x, and *A. sensitiva*) respectively. Intriguingly, previously only non-photosynthetic strains were isolated from *A. uniflora* root nodules^15^. It would thus be very interesting to investigate the nature of those present in its stem nodules and to compare its impact on nitrogen-fixation.

Given all the valuable information that can be gained from stem-nodulating legumes, we advocate reviving their study as this would significantly increase our understanding on the diversity of mechanisms underlying the nitrogen-fixing symbiosis in legumes. In turn, the acquired knowledge could help resolve some current research issues regarding the attempts to engineer nitrogen-fixation in non-legume crops. Notably, the photosynthetic activity of stem nodules and of some *Bradyrhizobium* strains could be an asset to sustain engineered nitrogen-fixation and diminish the possible competition for energy for C and N reduction.

## Material and methods

### Description of the collecting areas

To select collection sites of *Aeschynomene* and *Sesbania* species in Madagascar, we made use of general information about their distribution as described in the compendium "The Leguminosae of Madagascar" (Du Puy et al.^24^) and utilized precise location data of previous isolated specimens present in collections through the Global Biodiversity Information Facility (GBIF—https://www.gbif.org/) and the Tropicos database (https://www.tropicos.org/). This resulted in the definition of four collecting areas: (1) the region of Majunga including the vicinity of the Kinkony lake where many temporary to permanent marshes locally named « matsabory » are present. This area also comprises the plain of Marovoay where numerous rice fields form the main rice granary of Madagascar, (2) the Itasy region in the Center of Madagascar where the Itasy Lake and ricefields are present, (3) the Alaotra region in the East side of Madagascar that corresponds to a large basin containing the Alaotra Lake. The presence of extensive rice fields make this region the second rice granary of Madagascar, and (4) Nosy Be located towards the North of Madagascar where in the whole region rice fields are present. Expeditions to the four regions were made in April and May 2023, at the end of the rainy season that corresponds to the flowering period for most *Aeschynomene* and *Sesbania* species. Plants were collected in the above mentioned areas but also « en route » from ricefields, rivers and marshes present along the RN1, RN2 and RN4 national roads and secondary roads leading to the Itasy lake, the Alaotra lake and the region of Majunga, respectively.

### Plant and data collection

At each sampling location, the presence or receding of water in the aquatic ecosystem was recorded. The stem nodulation status of individuals for each species present in the population was examined and correlated to their positions relative to the flooding area. Whenever possible, three individuals of each species were chosen at random to determine their root nodulation status. Both stems and roots of these individuals were photographed in situ.

### Plant culture

Seeds collected in the field were dried at 34 °C for one week and used for plant cultivation when fresh material production was required. Seed scarification and plant growth in the greenhouse were performed as indicated^17^.

### Gene sequencing and sequence analysis

Genomic DNA was isolated from fresh leaves using the CTAB extraction method. The nuclear ribosomal internal transcribed spacer region (ITS: ITS1-5.8S rDNA gene-ITS2) and the chloroplast *matK* gene were amplified and sequenced as published in Chaintreuil et al. Additional ITS and *matK* sequences were retrieved from previous studies for *Aeschynomene* species^9,18,19^ and for *Sesbania* species^31^. To analyse sequence variations, sequences were aligned using Multalin (http://multalin.toulouse.inra.fr/multalin/multalin.html). ML phylogenetic tree reconstructions were obtained by aligning nucleotide sequences with the MUSCLE program that is incorporated in the MEGA X (v10.1.8) software. Aligned sequences were further processed in MEGA X using the maximum likelihood approach and the Kimura 2-parameter model with a 1000 × bootstrap (BS).

### Genome size estimation

Flow cytometry measurements were performed using fresh leaf material as described^20^. Genome size estimations were based on the measurements of three plants per accession using *Lycopersicum esculentum* (Solanaceae) cv « Roma » (2c = 1.99 pg) as the internal standard.

### Statement

Plant collection in Madagascar for purpose of this study was done in the frame of a convention research between the French IRD and Malagasy CNRE institutes (IRD Contract n°402075/00), with authorizations from the Malagasy Ministry of Scientific Research (licences n°114/23/MEDD/SG/DGGE/DAPRNE/SCBE.Re and 074-2023/MESupReS/SG/DGRS), and in compliance with the national Malagasy application of the Nagoya protocol. Jean-François Arrighi (PHIM Laboratory, IRD) undertook the formal identification of all the plant material collected in the field and used in this study. Plant material was frequently collected for germplasm conservation and production of voucher specimens. The latter were deposited at the public Herbarium of the CNARP Institute (https://cnarp.mg/) in Antananarivo (Madagascar) with deposition numbers indicated in the Supplementary Table 1.

### Supplementary Information

Supplementary Information.

## Data Availability

Data obtained in this study are listed in Tables S1 to S3, and the methods are given in Methods S1. The DNA sequences generated in this study were deposited in GenBank under accession numbers OR448903-OR448909 (nuclear *ITS*) and OR463925-OR463932 (chloroplastic *matK*).
