# Supplementary material for: Occurrence and diversity of stem nodulation in Aeschynomene and Sesbania legumes from wetlands of Madagascar
Source: Sci Rep. 2024 Feb 29;14:5024. doi: 10.1038/s41598-024-55247-7 (PMC10904833; doi:10.1038/s41598-024-55247-7)
Supplement: Supplementary file 1 — Supplementary Information. [file 41598_2024_55247_MOESM1_ESM.pdf]

Occurrence and diversity of stem nodulation in *Aeschynomene* and *Sesbania* legumes from wetlands of Madagascar

Faustin F. Manantsoa[1], Marrino F. Rakotoarisoa[2], Clémence Chaintreuil[3], Adamson T.E. Razakatiana[1], Frédéric Gressent[3], Marjorie Pervent[3], Mickaël Bourge[4], Martial D. Andrianandrasana[1], Nico Nouwen[3], Herizo Randriambanona[1], Heriniaina Ramanankierana[1], Jean-François Arrighi[3*]

[1]Laboratoire de Microbiologie de l'Environnement-Centre National de Recherches sur l'Environnement BP 1739 Fiadanana Antananarivo Madagascar. [2]Department of Ethnobotany and Botany, National Center for Applied Pharmaceutical Research, Antananarivo 101, Madagascar. [3]Plant Health Institute of Montpellier (PHIM), Univ Montpellier/IRD/INRAE/CIRAD/SupAgro, Campus de Baillarguet, 34398 Montpellier, France. [4]Cytometry Facility, Imagerie-Gif, Université Paris-Saclay, CEA, CNRS, Institute for Integrative Biology of the Cell (I2BC), 91198, Gif-sur-Yvette, France. *email: jean-francois.arrighi@ird.fr

a

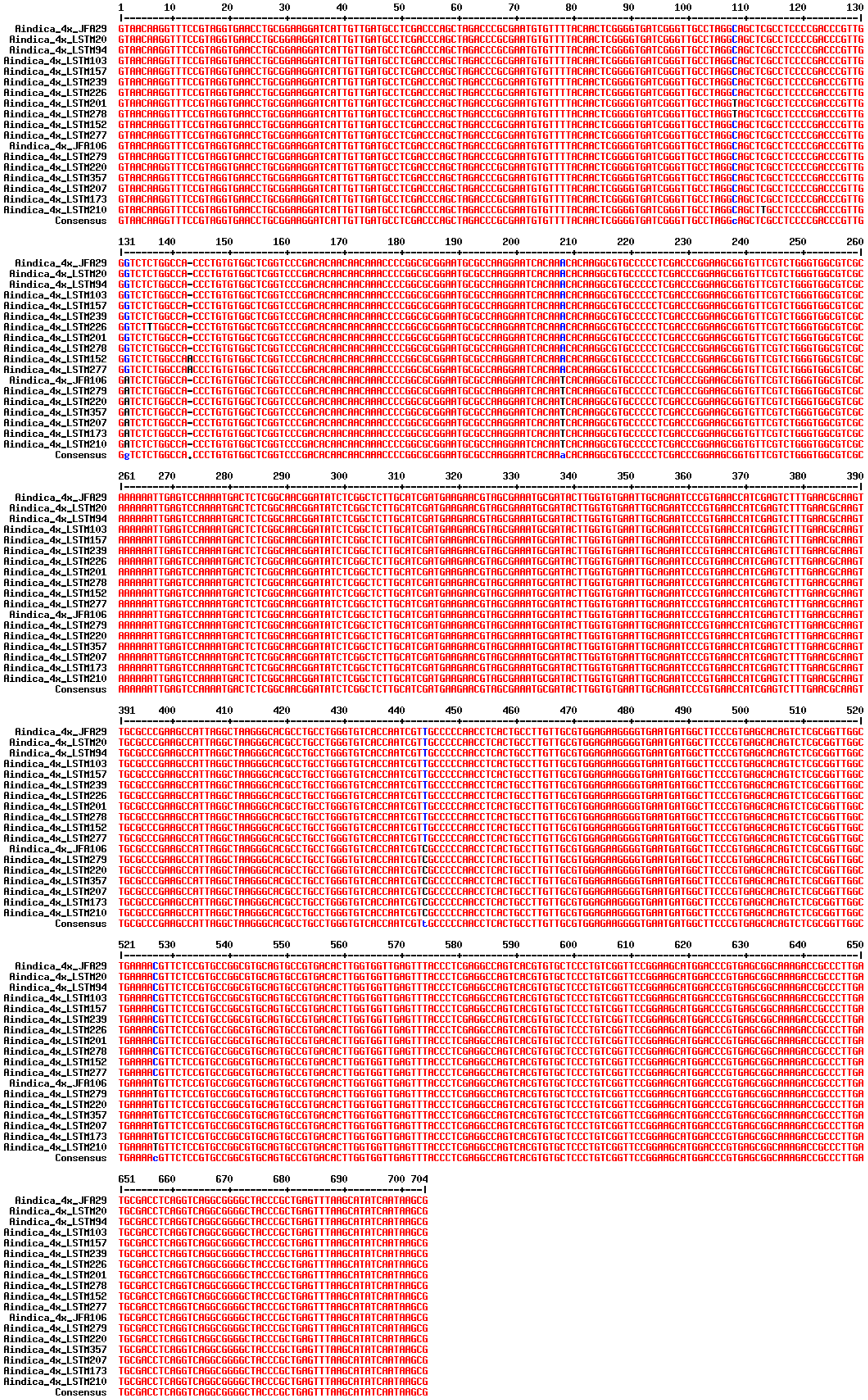
1 10 20 30 40 50 60 70 80 90 100 110 120 130
131 140 150 160 170 180 190 200 210 220 230 240 250 260
261 270 280 290 300 310 320 330 340 350 360 370 380 390
391 400 410 420 430 440 450 460 470 480 490 500 510 520
521 530 540 550 560 570 580 590 600 610 620 630 640 650
651 660 670 680 690 700 704
Aindica_4x_JFA29
Aindica_4x_LSTM20
Aindica_4x_LSTM94
Aindica_4x_LSTM103
Aindica_4x_LSTM157
Aindica_4x_LSTM239
Aindica_4x_LSTM226
Aindica_4x_LSTM201
Aindica_4x_LSTM278
Aindica_4x_LSTM152
Aindica_4x_LSTM277
Aindica_4x_JFA106
Aindica_4x_LSTM279
Aindica_4x_LSTM220
Aindica_4x_LSTM357
Aindica_4x_LSTM207
Aindica_4x_LSTM173
Aindica_4x_LSTM210
Consensus

b

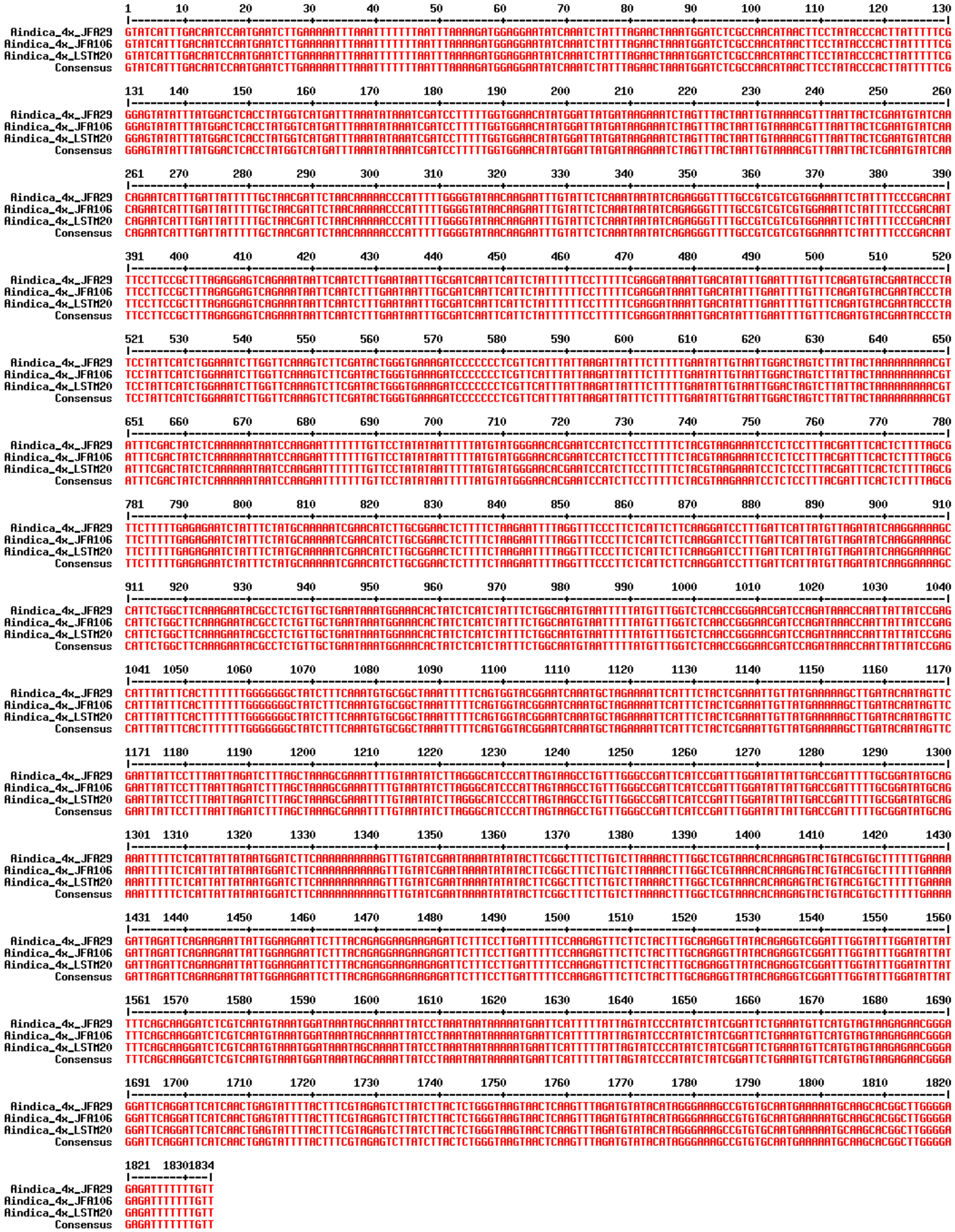

Supplementary Figure 1. Alignments of the nuclear *ITS* and chloroplastic *matK* gene sequences for *A. indica* 4x.(**a**) *ITS* sequence alignment for the specimens JFA29 and JFA106 using sequence data for *A. indica* 4x accessions generated by Chaintreuil *et al*. (2018). Note that the *ITS* sequences for the specimens JFA29 and JFA106 are different but correspond to the two main *ITS* variants found in *A. indica* 4x. (**b**) *matK* sequence alignment for the specimens JFA29 and JFA106 using sequence data for the *A. indica* 4x accession generated by Brottier *et al*. (2018). Note that the three *matK* sequences are identical.

a

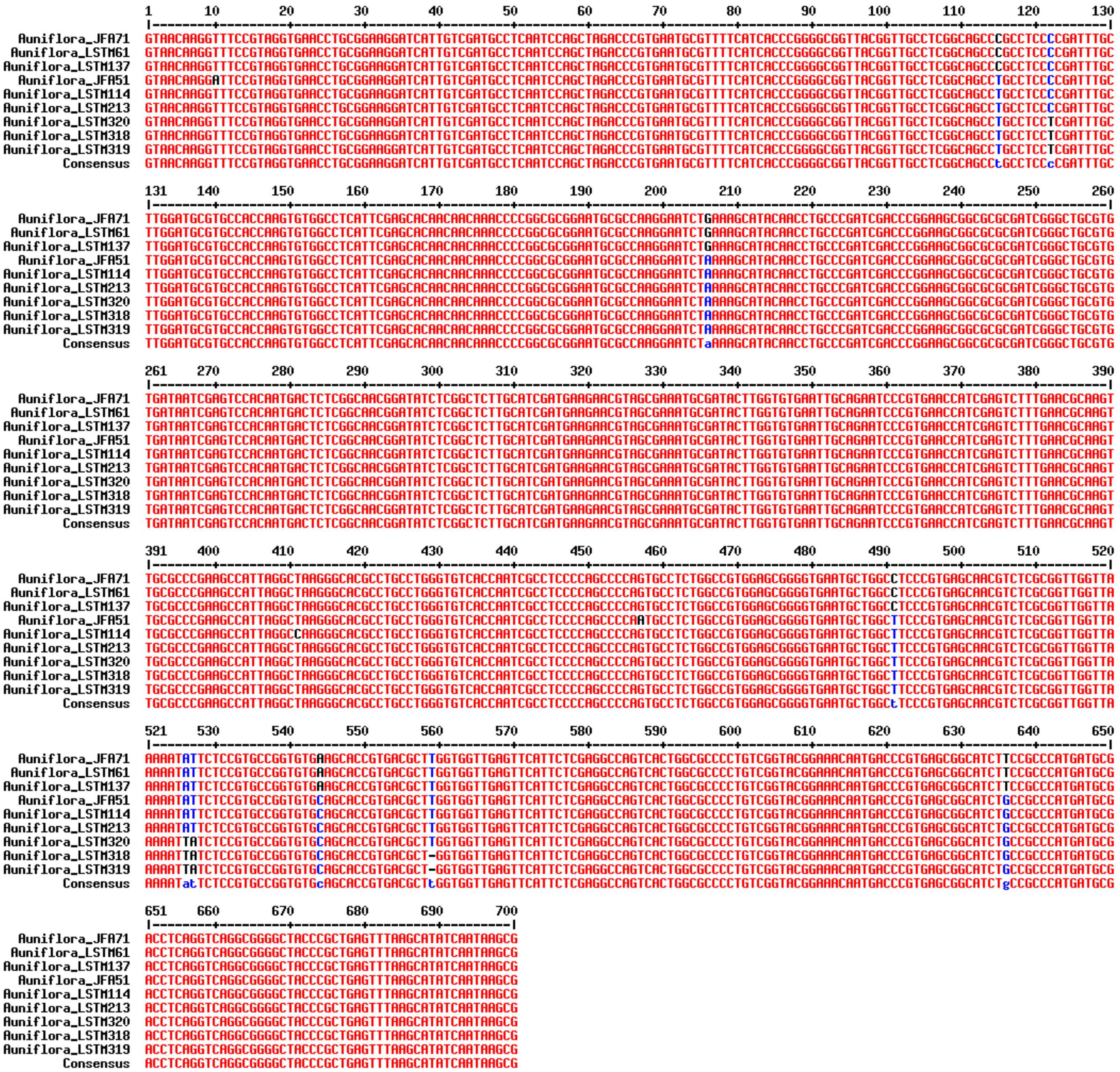
Auniflora_JFA71
Auniflora_LSTM61
Auniflora_LSTM137
Auniflora_JFA51
Auniflora_LSTM114
Auniflora_LSTM213
Auniflora_LSTM320
Auniflora_LSTM318
Auniflora_LSTM319
Consensus

b

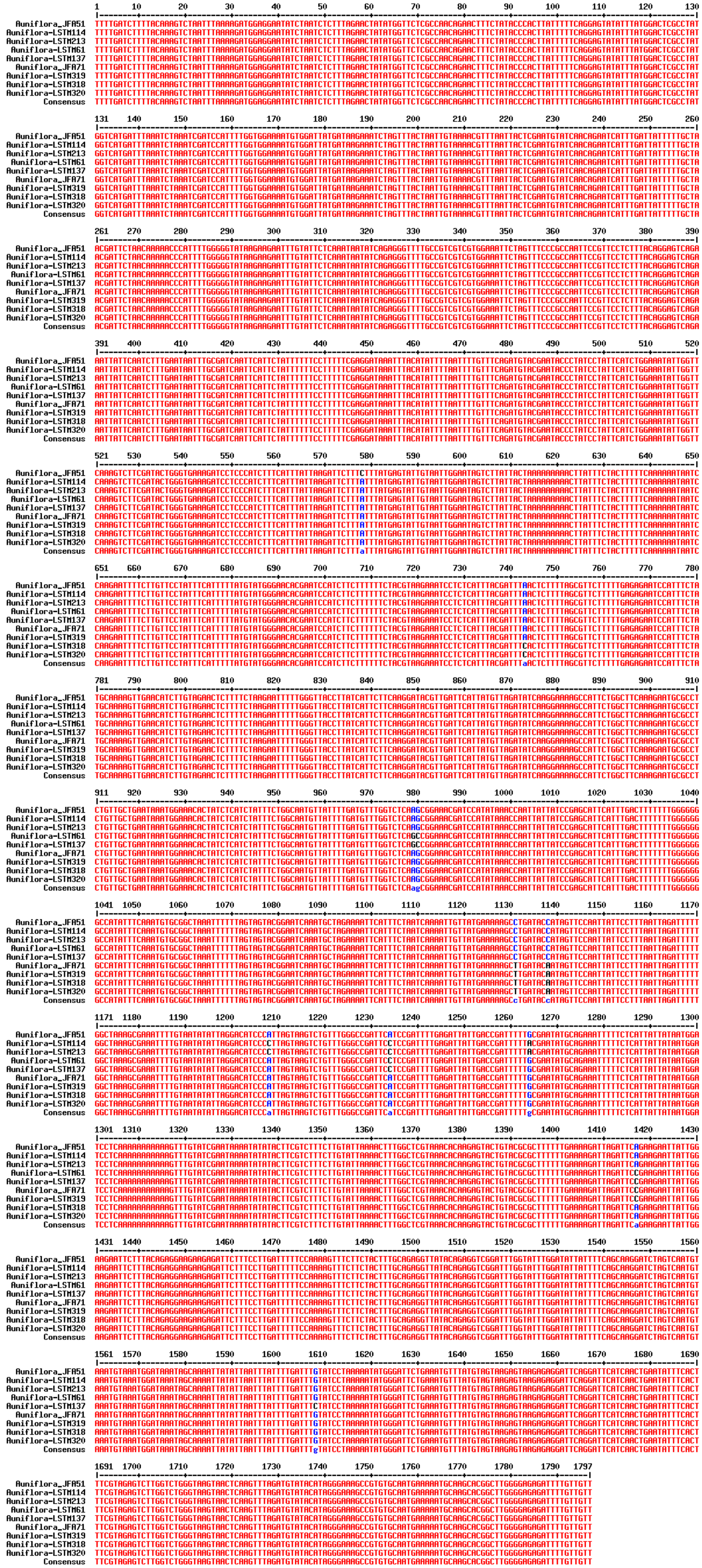

Supplemùentary Figure 2. Alignments of the (**a**) nuclear *ITS* and (**b**) chloroplastic *matK* gene sequences for *A. uniflora*. Sequence alignments for the specimens JFA51 and JFA71 using data for *A. uniflora* accessions generated by Chaintreuil *et al.* (2016). Note that the *ITS* sequence for the specimen JFA71 and the accessions LSTM61 and LSTM137, which have the small flower morphotype, share five SNPs.

a

```
                  1       10        20        30        40        50        60        70        80        90        100       110       120       130
Acristata_JFA34   GTAACAAGGTTTCCGTAGGTGAACCTGCGGAAGGATCATTGATGATGCCTCAATCTAGCTAGACCTGCGAATGCGTTAACTCACATGGGGCTGTTGTGGCTGTCCTGATAGCCTGCTTCCCCAAACAACC
Acristata_LSTM169 GTAACAAGGTTTCCGTAGGTGAACCTGCGGAAGGATCATTGATGATGCCTCAATCTAGCTAGACCTGCGAATGCGTTAACTCACATGGGGCTGTTGTGGCTGTCCTGATAGCCTGCTTCCCCAAACAACC
Consensus         GTAACAAGGTTTCCGTAGGTGAACCTGCGGAAGGATCATTGATGATGCCTCAATCTAGCTAGACCTGCGAATGCGTTAACTCACATGGGGCTGTTGTGGCTGTCCTGATAGCCTGCTTCCCCAAACAACC

                  131     140       150       160       170       180       190       200       210       220       230       240       250       260
Acristata_JFA34   GGGGCGTGGGCCACCCCGTGTGGCCTTGTCCCGGAACAACAACAAACCCCGGCGCGGAATGCGCCAAGGAACATACAAATGAACAACATGCCTGATCGACCCGGAAGCGGTGCTCGATATGGTTGCGTTG
Acristata_LSTM169 GGGGCGTGGGCCACCCCGTGTGGCCTTGTCCCGGAACAACAACAAACCCCGGCGCGGAATGCGCCAAGGAACATACAAATGAACAACATGCCTGATCGACCCGGAAGCGGTGCTCGATATGGTTGCGTTG
Consensus         GGGGCGTGGGCCACCCCGTGTGGCCTTGTCCCGGAACAACAACAAACCCCGGCGCGGAATGCGCCAAGGAACATACAAATGAACAACATGCCTGATCGACCCGGAAGCGGTGCTCGATATGGTTGCGTTG

                  261     270       280       290       300       310       320       330       340       350       360       370       380       390
Acristata_JFA34   TGATAATTGAGTCCATAACGACTCTCGGCAACGGATATCTCGGCTCTTGCATCGATGAAGAACGTAGCGAAATGCGATACTTGGTGTGAATTGCAGAATCCCGTGAACCATCGAGTCTTTGAACGCAAGT
Acristata_LSTM169 TGATAATTGAGTCCATAACGACTCTCGGCAACGGATATCTCGGCTCTTGCATCGATGAAGAACGTAGCGAAATGCGATACTTGGTGTGAATTGCAGAATCCCGTGAACCATCGAGTCTTTGAACGCAAGT
Consensus         TGATAATTGAGTCCATAACGACTCTCGGCAACGGATATCTCGGCTCTTGCATCGATGAAGAACGTAGCGAAATGCGATACTTGGTGTGAATTGCAGAATCCCGTGAACCATCGAGTCTTTGAACGCAAGT

                  391     400       410       420       430       440       450       460       470       480       490       500       510       520
Acristata_JFA34   TGCGCCCGAAGCCATTAGGCCAAGGGCACGCCTGCCTGGGTGTCACCAATCGCCTCCCTAATCCCGGTCCATGTTGACGTGGAGTGGGTTGAATGCTGGCTTCCCGTGAGCGATGTCTTGCGGTTGGTTG
Acristata_LSTM169 TGCGCCCGAAGCCATTAGGCCAAGGGCACGCCTGCCTGGGTGTCACCAATCGCCTCCCTAATCCCGGTCCATGTTGACGTGGAGTGGGTTGAATGCTGGCTTCCCGTGAGCGATGTCTTGCGGTTGGTTG
Consensus         TGCGCCCGAAGCCATTAGGCCAAGGGCACGCCTGCCTGGGTGTCACCAATCGCCTCCCTAATCCCGGTCCATGTTGACGTGGAGTGGGTTGAATGCTGGCTTCCCGTGAGCGATGTCTTGCGGTTGGTTG

                  521     530       540       550       560       570       580       590       600       610       620       630       640       650
Acristata_JFA34   AAAAAGTTCCCCGTGCTGGCGTGCGGTACCGTGACGCTGGGTGGTTGAGTTCATTCTCGAGGCCAGTCACGGTGCCCCTGTTGGCTCTGGAAATAGTGACCCGTGAGCGGCACTGACCGCCCATGATGCG
Acristata_LSTM169 AAAAAGTTCCCCGTGCTGGCGTGCGGTACCGTGACGCTTGGTGGTTGAGGTCATTCTCGAGGCCAGTCACGGTGCCCCTGTTGGCTCTGGAAATAGTGACCCGTGAGCGGCATTGACCGCCCATGATGCG
Consensus         AAAAAGTTCCCCGTGCTGGCGTGCGGTACCGTGACGCTgGGTGGTTGAGgTCATTCTCGAGGCCAGTCACGGTGCCCCTGTTGGCTCTGGAAATAGTGACCCGTGAGCGGCAcTGACCGCCCATGATGCG

                  651     660       670       680       690       700
Acristata_JFA34   ACCTCAGGTCAGGCGGGGCTACCCGCTGAGTTTAAGCATATCAATAAGCG
Acristata_LSTM169 ACCTCAGGTCAGGCGGGGCTACCCGCTGAGTTTAAGCATATCAATAAGCG
Consensus         ACCTCAGGTCAGGCGGGGCTACCCGCTGAGTTTAAGCATATCAATAAGCG
```

b

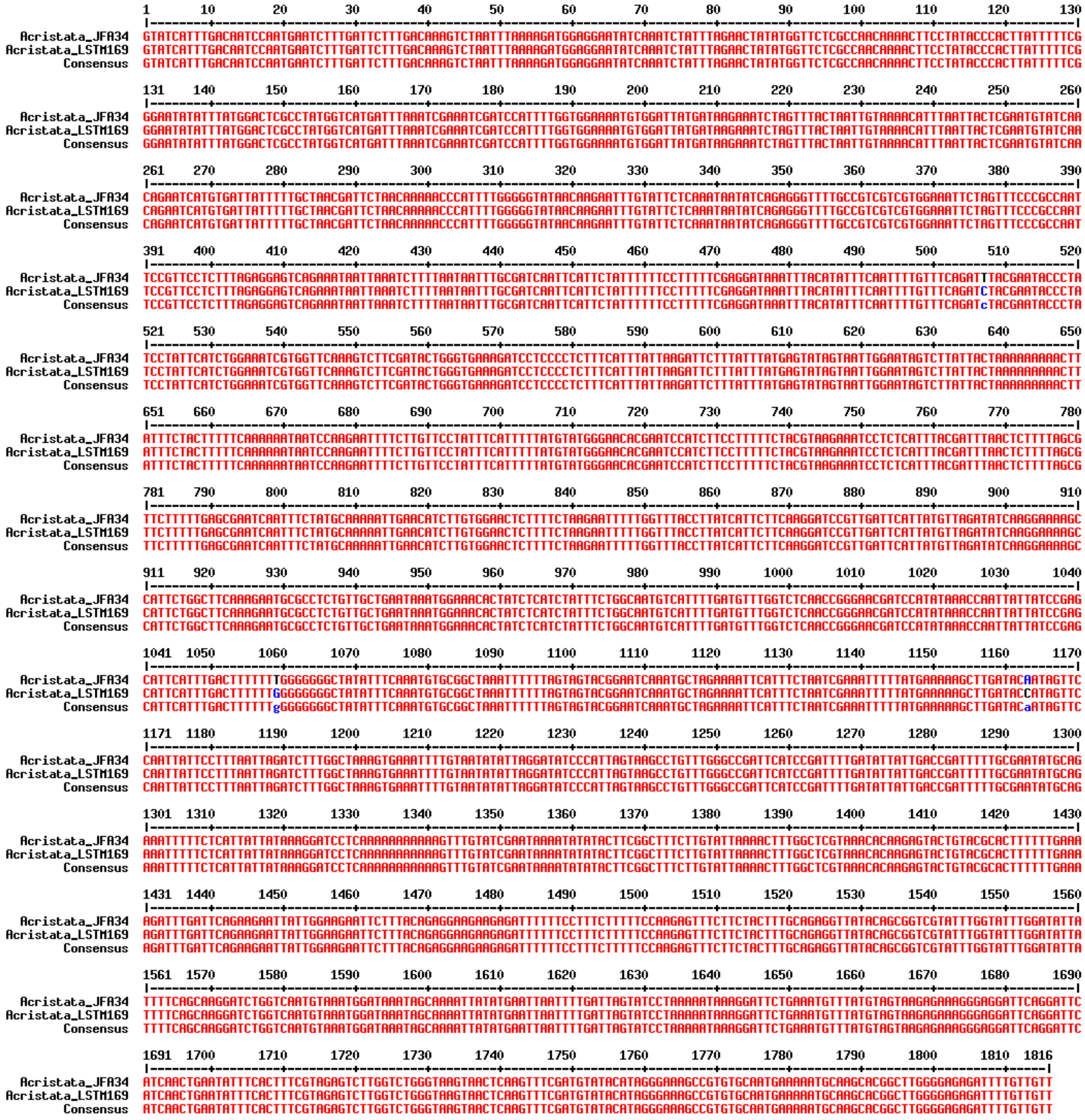

Supplementary Figure 3. Alignments of the (**a**) nuclear *ITS* and (**b**) chloroplastic *matK* gene sequences for *A. cristata*. Sequence alignments for the specimen JFA34 using data for *A. cristata* accession generated by Chaintreuil *et al*. (2016).

a

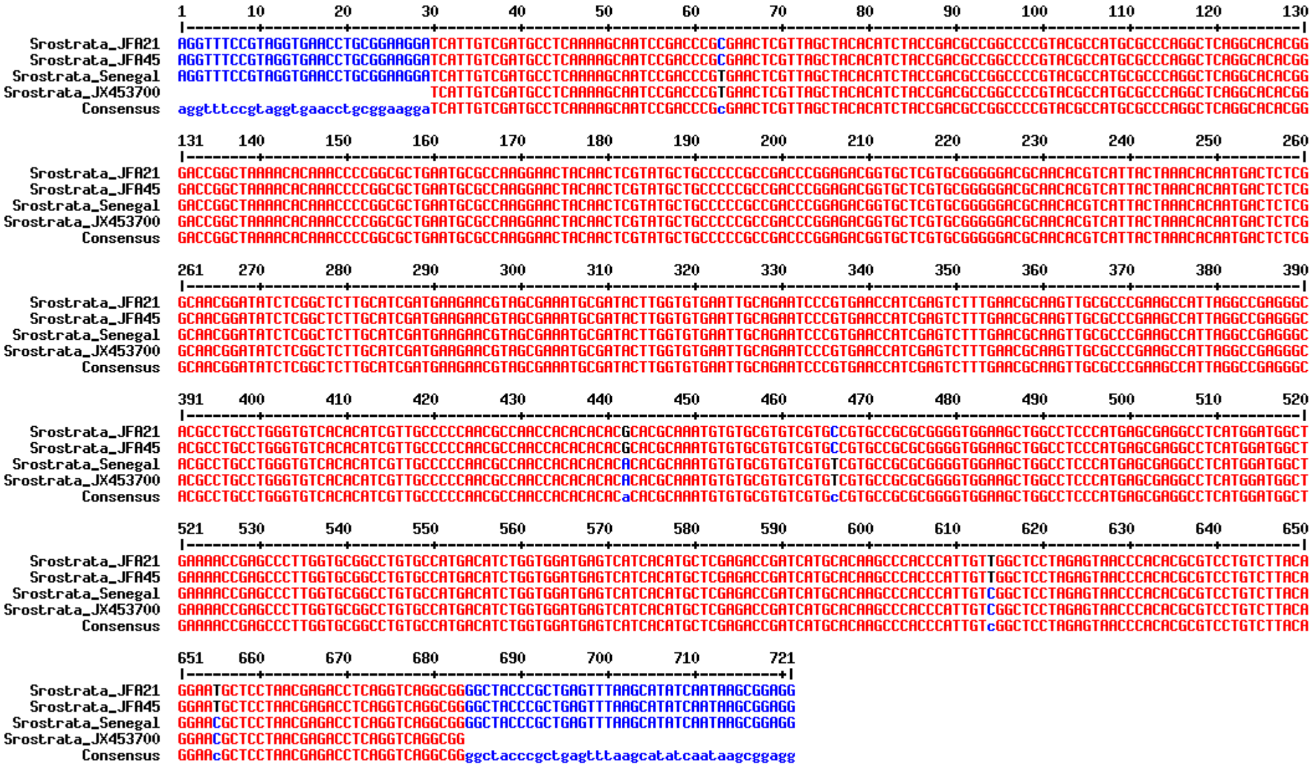
Srostrata_JFA21
Srostrata_JFA45
Srostrata_Senegal
Srostrata_JX453700
Consensus
1
721

b

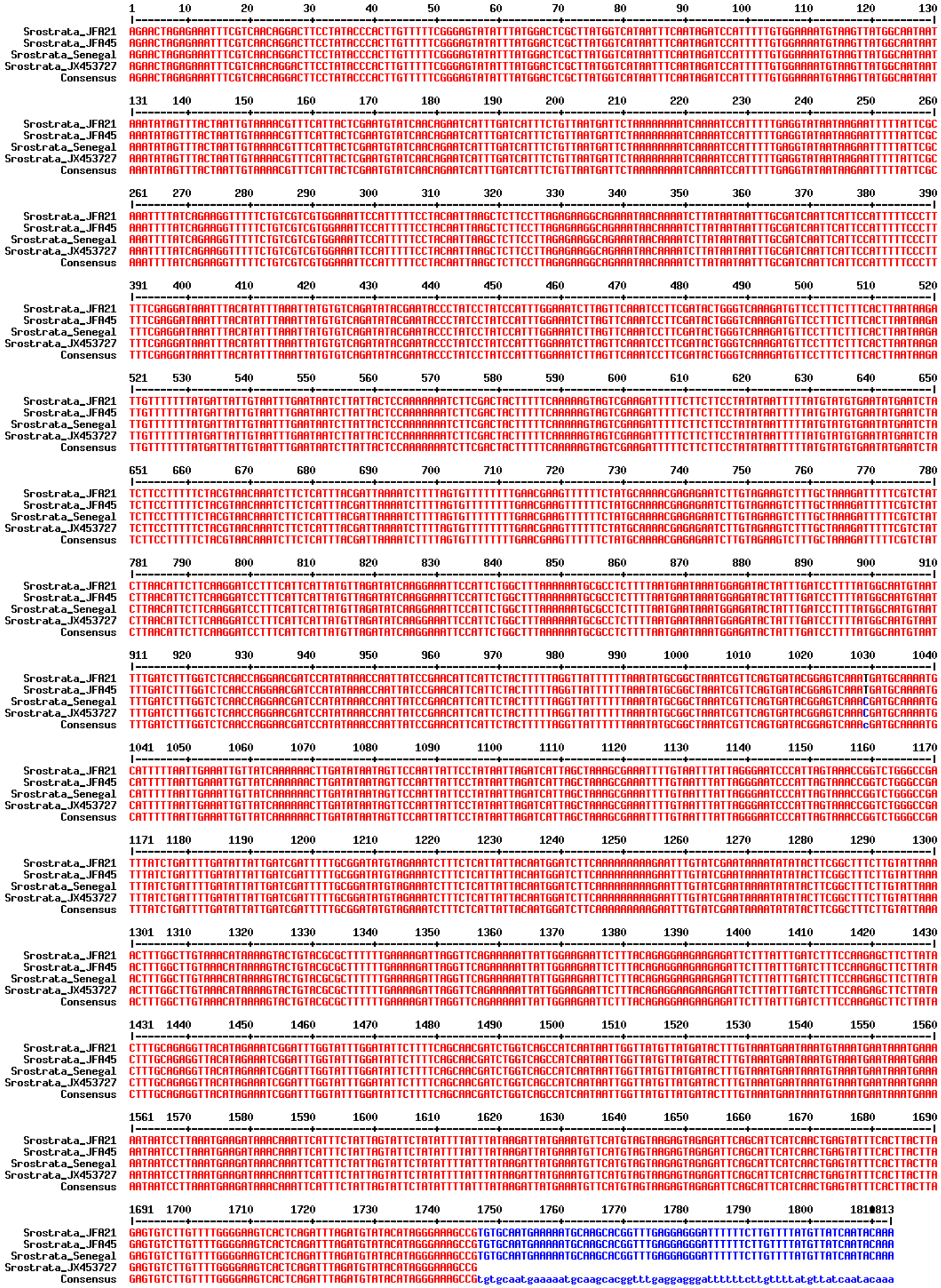

Supplementary Figure 4. Alignments of the (**a**) nuclear *ITS* and (**b**) chloroplastic *matK* gene sequences for *S. rostrata*. Sequence alignments for the specimens JFA21, JFA45 and an accession from Senegal (LSTM collection) using data generated by Farruggia *et al*. (2018). Note that the *ITS* sequence for the specimens from Madagascar differ in 5 SNPs when compared to those from Senegal.

Table S1 Accessions collected in this study, origin and characteristics

Species	Collection code	Sampling area	Location	GPS coordinates	Situation	Nodules	CNARP voucher
A. cristata	JFA34	RN4-Majunga	Analamany	S 15° 52' 08.3" E 45° 52' 45.9"	Marsh	Stem	RFM167
	JFA39	RN4-Majunga	Antangomenabevary	S 15° 58' 08.2" E 45° 59' 32.9"	Ricefield	Stem	
	JFA40	RN4-Majunga	Besokatra	S15 °52' 25.1" E 46° 09' 03.1"	River	Stem	RFM165
	JFA41	RN4-Majunga	Belemoka	S 15° 47' 59.5" E 46° 11' 07.1"	Marsh	Stem	
A. elaphroxylon	JFA88	RN2-Aloatra	Betoho	S 17° 46' 52.8" E 48° 26' 07.0"	Ricefield	Stem	RFM193
	JFA96	RN2-Aloatra	Ambongabe	S 17° 44' 44.7" E 48° 26' 49.3"	Ricefield	Root	RFM201
A. evenia	JFA2	RN4-Majunga	Andohatapenaka	S 18° 54' 02.2" E 47° 29' 46.8"	Ricefield	Stem	RFM157
	JFA3	RN4-Majunga	Mahitsy	S 18° 44' 22.0" E 47° 20' 21.2"	Ditch	Stem	
	JFA4	RN4-Majunga	Anjomoka	S 18° 40' 26.3" E 47° 17' 23.7"	Ricefield	Stem	
	JFA15	RN4-Majunga	Marofotroboka	S 16° 43' 11.2" E 47° 04' 05.3"	River	Stem	RFM163
	JFA17	RN4-Majunga	Tsaramandroso	S 16° 21' 22.8" E 47° 01' 32.7"	Marsh	Stem	
	JFA23	RN4-Majunga	Belobaka	S 15° 42' 27.0" E 46° 23' 29.5"	Marsh	Stem	
	JFA28	RN4-Majunga	Bekopaka	S 15° 41' 45.0" E 46° 22' 33.5"	Marsh	Root	
	JFA33	RN4-Majunga	Andrefamanangy	S 15° 57' 42.2" E 45° 57' 36.7"	Marsh	Stem	RFM166
	JFA35	RN4-Majunga	Analamany	S 15° 52' 08.3" E 45° 52' 45.9"	Marsh	Stem	
	JFA37	RN4-Majunga	Majunga - Besohaba marsh	S 15° 59' 26.8" E 45° 52' 34.6"	Ricefield	Stem	
	JFA47	RN4-Majunga	Majunga - Bevovoka marsh	S 16° 06' 03.2" E 46° 40' 52.2"	Ricefield	Stem	RFM175
	JFA50	RN4-Majunga	Bongomena	S 16°05' 11.4" E 46° 44' 38.2"	Ricefield	Stem	RFM178
	JFA54	RN4-Majunga	Antimimalandy crossing	S 16° 23' 36.8" E 47° 05' 47.9"	Ricefield	Stem	RFM182
	JFA56	RN4-Majunga	Carrière	S 16° 25' 38.0" E 47° 08' 07.4"	Marsh	Stem	RFM184
	JFA58	RN4-Majunga	Ampasambazaha	S 16° 56' 27.8" E 46° 49' 52.3"	Ricefield	Stem	RFM185
	JFA59	RN4-Majunga	Andriba	S 17° 36' 26.4" E 46° 55' 46.6"	Ricefield	Stem	RFM186

Table S1 Accessions collected in this study, origin and characteristics - following

A. evenia	JFA72	RN1-Itasy	Ampefy	S 19° 03' 25.6"<br>E 46° 44' 30.4"	Ricefield	Stem	JFA72
	JFA77	RN1-Itasy	Virgin Island	S 19° 04' 00.0"<br>E 46° 45' 48.9"	Ricefield	Stem	JFA77
	JFA86	RN2-Aloatra	Manakambahiny	S 18° 54' 22.2"<br>E 47° 58' 03.1"	Ricefield	Stem	RFM190
	JFA94	RN2-Aloatra	Camp Bandro	S 17° 38° 25.7"<br>E 48° 30' 18.2"	Ricefield	Stem	RFM199
	JFA98	RN2-Aloatra	Ambalabako	S 17° 50' 55.1"<br>E 48° 25' 09.8"	Ricefield	Stem	RFM203
	JFA100	RN2-Aloatra	Vodiala	S 17° 53' 04.1"<br>E 48° 15' 23.2"	Ricefield	Stem	RFM205
	JFA103	RN2-Aloatra	Ambohimangakely	S 18° 53' 31.4"<br>E 47° 36' 41.6"	Ricefield	Stem	
	JFA109	Nosy Be	Nosy Be - Andilana	S 13° 15' 14.6"<br>E 48° 11' 14.4"	Ricefield	Stem	
	JFA112	Nosy Be	Nosy Be - West road	S 13° 22' 37.3"<br>E 48° 13' 06.9"	Ricefield	Stem	
A. indica 4x	JFA27	RN4-Majunga	Majunga -<br>Amparihingidro	S 15° 42 '21.7"<br>E 46° 23' 16.1"	Marsh	Stem	RFM170
	JFA29	RN4-Majunga	Majunga -<br>Petite Plage marsh	S 15° 39' 34.7"<br>E 46° 20' 05.4"	Marsh	Stem	RFM169
	JFA106	Nosy Be	Nosy Be - East road	S 13° 27' 46.1"<br>E 48° 19' 16.1"	Ricefield	Stem	
	JFA107	Nosy Be	Nosy Be - East road	S 13° 17' 20.0"<br>E 48° 18' 38.5"	Ricefield	Stem	
A. schimperi	JFA1	RN1-Itasy	Antananarivo-<br>Tsimbazaza park	S 18° 55' 46.2"<br>E 47° 31' 31.7"	Garden	Root	
	JFA6	RN4-Majunga	Anjomoka	S 18° 40' 26.3"<br>E 47° 17' 23.7"	Ricefield	Root	RFM159
	JFA62	RN1-Itasy	Maharefo	S 19° 01' 07.9"<br>E 47° 11' 37.9"	Ricefield	Stem	JFA62
	JFA64	RN1-Itasy	Ankadimena	S 19° 56' 47.4'<br>E 49° 51' 45.4"	Ricefield	Stem	JFA64
	JFA68	RN1-Itasy	Ankazomasina	S 18° 57' 11.6"<br>E 46° 40' 06.0"	Ricefield	Stem	JFA68
	JFA70	RN1-Itasy	Ampefy	S 19° 03' 25.6"<br>E 46° 44' 30.4"	Ricefield	Stem	JFA70
	JFA74	RN1-Itasy	Mahiatrondro	S 19° 01' 15.8"<br>E 46° 43' 10.0"	Ricefield	Stem	JFA74
	JFA81	RN2-Aloatra	Sambaina	S 18° 53' 28.7"<br>E 47° 47' 20.5"	Ricefield	Stem	
	JFA84	RN2-Aloatra	Carion	S 18° 54' 37.6"<br>E 47° 42' 13.9"	Ricefield	Stem	RFM189
	JFA90	RN2-Aloatra	Ambotrasana	S 17° 43' 10.3"<br>E 48° 27' 24.7"	Ricefield	Stem	RFM195

Table S1 Accessions collected in this study, origin and characteristics - following

A. shimperi	JFA97	RN2-Aloatra	Ambalabako	S 17° 50' 55.1"<br>E 48° 25' 09.8"	Ricefield	Stem	RFM202
	JFA102	RN2-Aloatra	Ambohimangakely	S 18° 53' 31.4"<br>E 47° 36' 41.6"	River	Root	
A. sensitiva	JFA13	RN4-Majunga	Betsiboka bridge	S 16° 56' 26.1"<br>E 46° 57' 13.0"	Garden	Stem	RFM162
	JFA14	RN4-Majunga	Marofotroboka	S 16° 43' 11.2"<br>E 47° 04' 05.3"	River	Stem	
	JFA19	RN4-Majunga	Amboronozy plains	S 16° 09' 40.0"<br>E 46° 41' 02.8"	Marsh	Stem	
	JFA24	RN4-Majunga	Majunga - Belobaka marsh	S 15°42' 27.0"<br>E 46° 23' 29.5"	Marsh	Stem	RFM171
	JFA46	RN4-Majunga	Mandikanamana	S 16° 06' 55.9"<br>E 46° 43' 23.7"	Ricefield	Stem	RFM174
	JFA52	RN4-Majunga	Bemailaka	S 16° 20' 38.4"<br>E 46° 50' 40.0"	Marsh	Stem	RFM180
	JFA67	RN1-Itasy	Ankadinondry	S 19° 00' 16.9'<br>E 46° 27' 08.2"	Ricefield	Stem	JFA67
	JFA85	RN2-Aloatra	Manakambahiny	S 18° 54' 22.2"<br>E 47° 58' 03.1"	Ricefield	Stem	RFM191
	JFA87	RN2-Aloatra	Moramanga	S 18° 56' 42.0"<br>E 48° 13' 32.6"	Ricefield	Stem	RFM192
	JFA89	RN2-Aloatra	Tanambao-biampasika	S 17° 45' 51.6"<br>E 48° 26' 29.6"	River	Stem	RFM194
	JFA93	RN2-Aloatra	Ambatosoratra	S 17° 36' 20.9"<br>E 48° 30' 55.8"	Ricefield	Stem	RFM198
	JFA99	RN2-Aloatra	Ambalavato	S 17° 51' 18.3"<br>E 48° 18' 43.4"	Ricefield	Stem	RFM204
	JFA101	RN2-Aloatra	Andranovelana	S 18° 17' 51.0"<br>E 48° 16' 02.5"	Ricefield	Stem	RFM206
	JFA104	Nosy Be	Nosy Be - South East	S 13° 22' 47.4"<br>E 48° 19' 53.9"	Ricefield	Stem	
A. uniflora	JFA51	RN4-Majunga	Bongomena	S 16° 05' 11.4"<br>E 46° 44' 38.2"	Ricefield	Stem	RFM179
	JFA53	RN4-Majunga	Bemailaka	S 16° 20'38.4"<br>E 46° 50' 40.0"	Marsh	Stem	RFM181
	JFA71	RN1-Itasy	Ampefy	S 19° 03' 25.6"<br>E 46° 44' 30.4"	Ricefield	Stem	JFA71
	JFA80	RN1-Itasy	Soavinandriana	S 19° 11' 12.8"<br>E 46° 45' 50.4"	Ricefield	Root	JFA80
	JFA108	Nosy Be	Nosy Be - Ampasindava	S 13° 16' 17.4"<br>E 48° 16' 51.8"	Ricefield	Stem	
	JFA111	Nosy Be	Nosy Be - West road	S 13° 22' 37.3"<br>E 48° 13' 06.9"	Ricefield	Stem	
S. rostrata	JFA21	RN4-Majunga	Amboromalandy	S 16° 07' 08.8"<br>E 46° 45' 06.1"	Marsh	Stem	RFM164
	JFA45	RN4-Majunga	Ambalanomby	S 16° 06' 28.3"<br>E 46° 39' 16.4"	Ricefield	Stem	

Table S2 Nuclear 2C DNA amounts obtained by flow cytometry.

Species	Code	2C DNA content (pg)	Origin	Reference
A. cristata	JFA34	1,81 ± 0.00	Madagascar	This study
	ILRI16880/LSTM169	1.92 ± 0.02	CAR	Chaintreuil *et al*. (2016)
A. indica 4x	JFA29	1,72 ± 0.02	Madagascar	This study
	JFA106	1,67 ± 0.01	Madagascar	This study
	CIAT17341/LSTM20	1.71 ± 0.07	Thailand	Arrighi *et al*. (2014)
A. uniflora	JFA51	2.78 ± 0.02	Madagascar	This study
	JFA71	2.73 ± 0.01	Madagascar	This study
	LSTM319	2.70 ± 0.04	Madagascar	Chaintreuil *et al.* (2016)
S. rostrata	JFA21	2.57 ± 0.02	Madagascar	This study
	JFA45	2.58 ± 0.02	Madagascar	This study
	Senegal	2.59 ± 0.01	Senegal	This study

Table S3 GenBank numbers for the sequences generated in this study.

Species	Accession	*ITS*	*matK*
A. cristata	JFA34	OR448910	OR463928
A. indica 4x	JFA29	OR448906	OR463929
A. indica 4x	JFA106	OR448907	OR463930
A. uniflora	JFA51	OR448908	OR463931
A. uniflora	JFA71	OR448909	OR463932
S. rostrata	Senegal	OR448903	OR463925
S. rostrata	JFA21	OR448904	OR463926
S. rostrata	JFA45	OR448905	OR463927
